# Investigating the Potential of Conjugated Selenium Redox Folic Acid as a Treatment for Triple Negative Breast Cancer

**DOI:** 10.3390/antiox9020138

**Published:** 2020-02-05

**Authors:** Soni Khandelwal, Mallory Boylan, Gilbert Kirsch, Julian E. Spallholz, Lauren S. Gollahon

**Affiliations:** 1 Department of Nutritional Sciences, Texas Tech University, Lubbock, TX 79409, USA; soni.khandelwal87@gmail.com (S.K.); malloryboylan1@gmail.com (M.B.); spallholz@aol.com (J.E.S.); 2 University of Lorraine, 57070 Metz, France; gilbert.kirsch@univ-lorraine.fr; 3 Department of Biological Sciences, Texas Tech University, Lubbock, TX 79409, USA

**Keywords:** Selenofolate, Folic Acid, selenium, superoxide, TNBC cells, MDA-MB-468 cells, mammary epithelial cells, HME50-5E cells, targeted cancer therapy

## Abstract

Previous studies have demonstrated that redox selenium compounds arrest cancer cell viability in vitro through their pro-oxidative activity by generating superoxide (O_2_^•−^). Currently, there are no efficacious treatment options for women with Triple Negative Breast Cancer (TNBC). However, the association between the over-expression of the Folate Receptor Alpha (FRA) in TNBC and other cancer cells, has led to the possibility that TNBCs might be treated by targeting the FRA with redox selenium covalent Folic Acid conjugates. The present study reports the synthesis of the redox active vitamer, Selenofolate, generating superoxide. Superoxide (O_2_^•−^) catalytic generation by Selenofolate was assessed by an in vitro chemiluminescence (CL) assay and by a Dihydroethidium (DHE) in vivo assay. Cytotoxicity of Selenofolate was assessed against the TNBC cell line MDA-MB-468 and an immortalized, mammary epithelial cell line, HME50-5E. Cytotoxicity of Selenofolate was compared to Folic Acid and sodium selenite, in a time and dose dependent manner. Selenofolate and selenite treatments resulted in greater inhibition of MDA-MB-468 cell proliferation than HME50-5E as evaluated by Trypan Blue exclusion, 3-(4,5-dimethylthiazol-2-yl)-2,5-diphenyltetrazolium bromide (MTT) metabolic assay and Annexin V apoptosis assays. Folate receptor alpha (FRA) protein expression was assessed by Western blotting, with the experimental results showing that redox active Selenofolate and selenite, but not Folic Acid, was cytotoxic to MDA-MB-468 cells in vitro, suggesting a possible clinical option for treating TNBC and other cancers over-expressing FRA.

## 1. Introduction

Reduced Folic Acid plays a coenzyme role in the synthesis of purines and pyrimidines for DNA, and other cellular methylation reactions [1]. Dietary Folic Acid is absorbed by intestinal cells and released in its coenzyme form, 5-methyl-tetrahydrofolate (5-MTHF) into the bloodstream, the form which it is taken up by all cells [2] through one or more of four 5-MTHF Folic Acid receptor isoforms; (Folate Receptor alpha, FRA; beta, FRB; gamma, FRG; or delta, FRD). Uptake of Folic Acid into cells through these receptors maintains Folic Acid homeostasis, which is essential for cellular division and proliferation [3,4]. FRA is a 38 kDa single chain glycosylphosphatidylinositol (GPI)-anchored cell surface cysteine-rich glycoprotein [5] that was first discovered by Coney et al. in 1991 [6] in the human ovarian carcinoma cell line IGROV-1, binding to 5-MTHF. The FRA facilitates vitamin entry into the cell [7]. Its protein synthesis is through expression of the FOLR1 gene [8]. FRA is most predominantly expressed on the apical surface of a subset of epithelial tissue cells [9,10] including lung, parotid, thyroid, and kidney [11]. Of these, FRA is most highly expressed on kidney cells [12]. Various cancers are known to over-express FRA and have a greater affinity for Folic Acid [12,13,14] than do normal cells, due to their rapid and constitutive proliferation. Because of this higher FRA expression and affinity for Folic Acid in cancer cells versus normal tissues, (excluding kidney), Folic Acid conjugates have become applicable treatment strategies for targeting ovarian, endometrial, colorectal, kidney, lung and breast cancers with covalent florescent tags [15], in addition to ^99m^Tc-etarfolatide [16,17] to enhance metastasized ovarian surgical cancer treatment. More recently a folate-drug conjugate of Vinblastine, Vintafolide, showed promising preclinical activity in Triple Negative Breast Cancer (TNBC) xenograft models [18] and was taken through Phase 3 clinical trials for treating human ovarian cancer where it eventually failed because it did not demonstrate efficacy on the prespecified outcome of progression-free survival (PFS) in women with platinum-resistant ovarian cancer [19]. We have had very good success in the synthesis of Transferrins [20] and monoclonal antibodies [21] with redox selenium conjugates in in vitro models that have demonstrated significant arrest of cell division of leukemias, Her2/neu [22] and TNBC cells [21]. Based on our prior redox selenium conjugate success coupled with the fact that TNBC does not adequately benefit from conventional therapies, tumor-associated FRA appears to be a therapeutic target for many cancers over-expressing the FRA. To that end, we hypothesized that a covalently conjugated Folic Acid with a redox selenium adduct, would show significant cytotoxicity in cancer cells versus normal cells. This conjugated species is herein referred to as Selenofolate.

## 2. Materials and Methods

### 2.1. Materials

DMEM high glucose (Catalog # 1965-092), Fetal Bovine Serum (Catalog #10082-147), 1% Penicillin-Streptomycin (Catalog #15-140-122), were purchased from Life Technologies, Gibco (Carlsbad, CA, USA). pH test strips (Catalog #8882-1) were purchased from Ricca Chemical Company (Arlington, TX, USA). PVDF membranes (Catalog #1620177), Non-Fat Milk Protein (Catalog #1706404), 3–20% Tris-Glycine Polyacrylamide Gel (Catalog #456-1096) and 2× native PAGE sample buffer (Catalog #161-0738) were purchased from BIO-RAD (Hercules, CA, USA). Mammary Epithelial Cell Media (Catalog #50-306-176) was purchased from PromoCell (Heidelberg, Germany). Trypsin (Catalog #30-2101) was purchased from ATCC (Manassas, VA, USA). Rabbit Anti-Mouse IgG H&L HRP (Catalog #ab6728) was purchased from Abcam (Cambridge, UK). A 0.22 µM filter (Catalog #SLGV033RS), Anti-β-Actin Antibody (Catalog #MAB1501) and Accutase (Catalog #SCR005) from EMD Millipore (Burlington, MA, USA). Corning^®^ cell culture 75 cm^2^ (Catalog #CLS430641) and 25 cm^2^ (Catalog #CLS430639) vented cap flasks, Corning^®^ Costar^®^ TC-Treated Multiple Well Plates (Catalog #CLS3527), Corning^®^ Costar^®^ TC-Treated Multiple Well Plates (Catalog #CLS3548), Sodium selenite (Catalog #14485), 3-(4,5-dimethylthiazol-2-yl)-2,5-diphenyltetrazolium bromide (MTT) assay (Catalog #M2128), Superoxide Dismutase (Catalog #S7571), Bovine Erythrocytes (Catalog #C1345-1G), Dihydroethidium (DHE) (Catalog #D7008), Lucigenin (Catalog #M8010-5G) were purchased from Sigma-Aldrich (St. Louis, MO, USA). RIPA lysis buffer (Catalog #89900), Anti-Folate Receptor Alpha antibody (Catalog #MA5-23917), SuperSignal™ West Femto Maximum Sensitivity Substrate (Catalog #34095), Gamma Globulin (Catalog #23212), BCA assay kit (Catalog #23225), MitoTracker™ Red CMXRos (Catalog #M7512), nd Annexin V-FITC Apoptosis Detection Kit (Catalog #BMS500FI-300) were purchased from ThermoFisher Scientific (Waltham, MA, USA). The TNBC MDA-MB-468 cells were obtained from ATCC and the immortalized HME50-5E cells were derived by Dr. Gollahon from normal tissue outside the area of surgical resection of an invasive ductal carcinoma.

### 2.2. Synthesis of Selenofolate

For synthesis of 2-selenocyanoethanol, a mixture of 0.01 mol of 2-chloroethanol (0.805 g) and 0.01mol of KSeCN (1.44 g) in 50mL of acetonitrile was refluxed for 4 h. Acetonitrile was then evaporated under vacuum. The residue was taken into 100 mL of water and extracted with 3 × 50 mL of diethylether. After drying with MgSO_4_, filtering and evaporation of the solvent, the residue (~1.4 g) corresponding to 2-selenocyanoethanol, was used without further purification for H1NMR: 4.1 ppm; triplet 2H, 3.8 ppm; triplet 2H.

Synthesis of 2-selenocyan folate Selenofolate was achieved by mixing 1 mmol (441 mg) of Folic Acid dissolved in 100 mL DMSO with 2 mL of pyridine, 1 mmol of dicyclohexylcarbodiimide (206 mg) and 1 mmol of 2-selenocyanoethanol (150 mg) for 72 h at room temperature. The DMSO solution was then poured into 500 mL of a mixture of 1:1 diethylether–acetone. The mixture was stirred well until the Selenofolate commenced precipitating. Filtration washing with acetone and drying in a desiccator yielded 345 mg of folic acid as a reddish powder (~60%). The Selenofolate can then be boiled in acetone to eliminate all DMSO traces. Purity of the folic acid was checked by microanalysis.Folic Acid The chemical reaction for synthesis of selenocyanethanol and Selenofolate is included in Supplemental Materials Figure S1.

**Figure 1:**
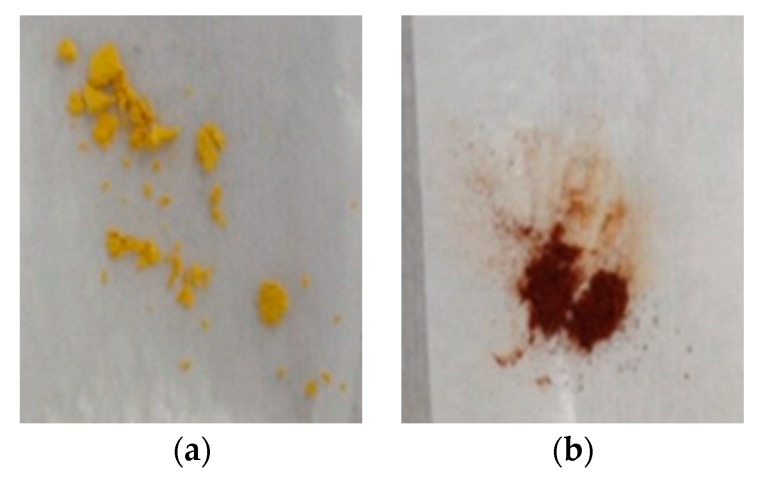
Yellow Folic Acid (**a**) and red Selenofolate (**b**).

**Figure 2:**
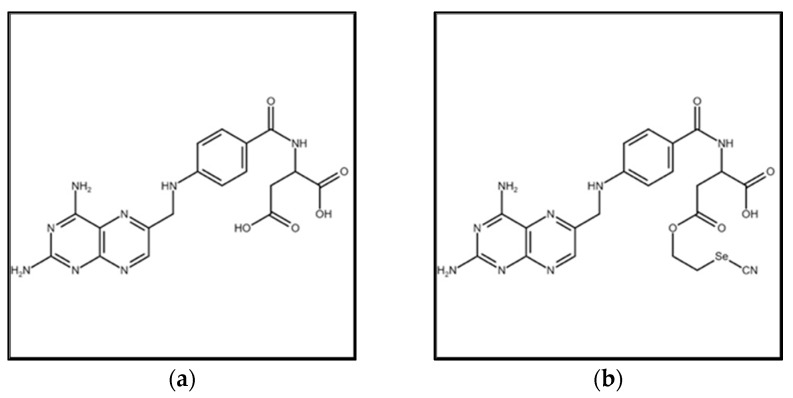
Structure of Folic Acid (**a**) and Selenofolate (**b**).

The solid precipitate containing Selenofolate was filtered and washed with ether and acetone. The yield of the dried, red Selenofolate was approximately 60% and was used without further purification. Dried Folic Acid (yellow) and the converted Selenofolate (red) are shown in Figure 1, with the corresponding chemical structures of Folic Acid and Selenofolate shown in Figure 2. 

### 2.3. Detection of Superoxide Generation In Vitro by Folic Acid and Selenofolate

**Figure 3:**
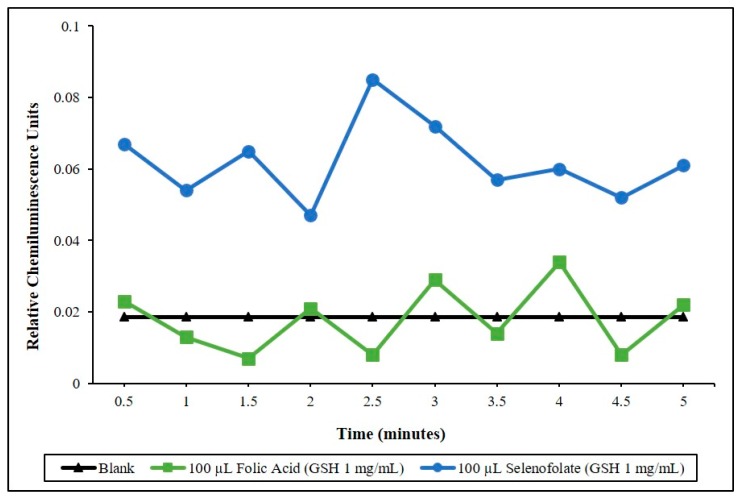
Time dependent superoxide generation as a function of lucigenin chemiluminescence. Chemiluminescence (CL) was measured for blank, Folic Acid and Selenofolate, 100 µL of Selenofolate = 70 µg of Se. Real time (CL) assay in 30 s integrations.

Lucigenin (Bis-*N*-Methylacridinium Nitrate) was used to detect superoxide (O_2_^•−^) generation in a chemiluminescent (CL) assay which consisted of PBS pH 7.4, reduced GSH (1 mg/mL) and 20 ug/mL of Lucigenin as previously described [23]. A Turner TD-20e Luminometer (Turner Designs Inc., Mountain View, CA, USA), connected to a 37 °C circulating water bath was used to analyze the O_2_^•−^ superoxide, the generation of which is proportional to the chemiluminescent light detected. Using the source of electrons for the generation of O_2_^•−^ is from the GSH catalyzed and oxidized by selenite and organic selenides [24]. CL measurements of the blank cocktail, Folic Acid and Selenofolate catalysis were taken by adding different volumes (0–100 µL) of a stock Folic Acid (5 mg/mL) and stock Selenofolate (5 mg/mL) solution to 500 µL of the CL cocktail at 37 °C. Data was integrated over 5 min in 30 s increments; *n* = 10. The mean CL values of 3 separate assays; control cocktail (blank), 100 μL of Folic Acid and Selenofolate is shown in Figure 3 in the Section 3.

### 2.4. Cell Culture

Dulbecco’s Modified Eagle’s Media (DMEM) with high glucose and supplemented with 10% fetal bovine serum and 1% Penicillin-Streptomycin was used to support MDA-MB-468 cells were cultured in 75 cm^2^ tissue flask. Cells were cultured at 37 °C under humid conditions in a 5% CO_2_ incubator for 2–3 days. Cell media was changed twice per week. At 75–85% confluence, cells were washed with PBS, pH 7.4 and trypsinized with 5 mL of 0.25% (*v/v*) trypsin/0.03% EDTA. HME50-5E cells were also cultured in 75 cm^2^ tissue culture flasks containing Mammary Epithelial Cell Media as previously described [25]. Cells were incubated for 7 to 8 days at 37 °C under humid conditions in the same 5% CO_2_ incubator. Media was changed 3–4 times per week. At 60–70% confluence, cells were washed with ice cold PBS and trypsinized with 5 mL Accutase™. 

Optimization of cell growth densities was plotted against time for 7 days to determine and accurate log growth for subsequent experiments. The Folic Acid and Selenofolate experimental treatments were determined from the log growth curves generated by seeding 5000; 10,000; 20,000; 40,000 and 100,000 cells/well in 48-well plates. 

### 2.5. Western Analysis of FRA Protein Expression

For Western analysis of FRA protein expression, 10^6^ MDA-MB-468 and HME50-5E cells were seeded onto T-25 flasks and allowed to attach overnight. After 24 h, cells were treated with 50, 75, or 100 µM of Folic Acid, Selenofolate or PBS and incubated for 3 days. On day 3, cells and supernatants were collected in a 15 mL tube and washed with ice cold 1× PBS. The tube was centrifuged at 4000× *g* for 4–5 min, after which the media was carefully aspirated using a Pasteur pipette. Two hundred µL of RIPA lysis buffer was added to the cell pellets, and the samples were kept at −80 °C for 2 h, then thawed to generate better yields. To collect the very adherent HME50-5E cell lysates, the flasks were carefully broken with a hammer and cells were scrapped off using a cell scraper, collected and put in ice for 5 min. The HME50-5E lysates were then passed through a 20-gauge needle and kept on ice for another 5 min. All samples were kept on ice for an additional 15 min before centrifugation for 15 min at 12,000× *g* at 4 °C. Total protein concentration in the cleared lysates was determined using the bicinchoninic acid (BCA) assay according to the manufacturer’s instructions. After protein concentration quantitation, 50 µg of total protein was separated on 8% denaturing polyacrylamide gels and electroblotted to PVDF membranes. Membranes were blocked for 1 h in a solution of PBS containing 0.05% Tween-20 (PBST) and 5% non-fat dry milk protein. Gels were then incubated overnight with an anti-FRA antibody diluted to 2 µg/mL in PBST or anti-β-actin antibody diluted 1:1000 in PBST containing 1% non-fat milk protein. After 24 h, the membrane was washed 3 times for 15 min each in PBST, incubated for 1 h with horse-radish peroxidase conjugated with rabbit anti-mouse IgG diluted 1:10,000 in PBST, and washed once in PBST for 15 min. Antibody complexes bound with HRP were visualized using the SuperSignal™ West Femto Maximum Sensitivity Substrate.

### 2.6. Folic Acid and Selenofolate Treatments

All experimental controls, Folic Acid and Selenofolate treatments were performed under aseptic cell culture conditions in a HEPA environment. Exponentially growing MDA-MB-468 and HME50-5E cells were harvested from flasks and viability determined by Trypan Blue exclusion. Cells were plated at 40,000 cells/well into 48-well and cultured for 5 days prior to treatments with a medium change on day 3. Treatments commenced on day 5 with the addition of fresh culture media.

Control cells were treated with PBS alone, while MDA-MB-468 cells and HME50-5E cells were treated with Folic Acid or Selenofolate at final concentrations of 1–100 µM (0.08–8 µg Se) in PBS. Due to its known toxicity to cells, sodium selenite [21,22,23,24] was also used to treat both cell lines at final concentrations of 20 µM/well (10 µg Se). All experiments were performed in biological and technical triplicates in 48-well plates and analyzed on consecutive days (0–7), for cytotoxicity and cell viability.

### 2.7. Photographic Assessment of Control and Treated Cell Morphology

Cells were treated on Day 0 with PBS, 20 µM Selenite (10 µg Se), 100 µM Folic Acid and 100 µM Selenofolate (8 µg Se). Control, Folic Acid, Selenofolate and Selenite treated cell were photographed without Trypan Blue on consecutive days 1–6 post-treatments using an EVOS XL Core (Life Technologies, Carlsbad, CA, USA) microscope under phase contrast, to record any morphological changes.

### 2.8. Cell Viability Analyzed by Trypan Blue Exclusion

Control and treated cell viability were determined by Trypan Blue exclusion on a Beckman Coulter Vi-Cell Viability Analyzer (Beckman Coulter, Inc. Model VI-CELL SGL, Indianapolis, IN, USA). Trypan Blue determines the integrity of the plasma membrane. Cells with unperturbed membranes (and thus clear of the dye) are considered viable. Forty-thousand cells/well were seeded in 48-well flat-bottom plates. Cell volumes were adjusted with media and the cells were cultured for 5 days prior to treatments. Media was changed on day 3 post-seeding. Cells were treated with PBS alone while 5–100 µM of either Folic Acid or Selenofolate treated cells in triplicates over assayed over 7 days. Control cells were treated with 4, 10, 20 and 40 µM (2–20 µg Se) of selenite.

Cells were treated for 7 days. Each collection day, 500 µL of media was used to wash cells for 5 min and 200 µL of 0.025% trypsin-EDTA was then added to harvest the adherent cells. After 5 min of incubation, another 200 µL of trypsin was added to the wells for cell collection. Cells were analyzed with the Beckman Coulter Vi-Cell Viability Analyzer for cell viability. 

### 2.9. Cell Viability as Measured by the MTT Assay

MTT is an assay developed to determine viability based on cellular metabolic activity. Cells were treated on day 5 post-seeding with the various concentrations of Folic Acid equivalent to Selenofolate for 6 days. Control treatments included 20 µM selenite/well and PBS alone. Phenol red-free DMEM—high glucose, + 10% FBS and MTT at 5 mg/mL was sterilized through a 0.22-micron filter, protected from light exposure, and used immediately. A 10% (*v/v*) solution of 50 µL of MTT (5 mg/mL) was added to each well and samples were incubated at 37 °C for 3 h. Following incubation with the MTT, 500 µL formazan solubilization solution and acidified isopropanol (0.1N HCl) with 10% Triton X-100, was added in each well. To assess metabolic activity, a Cytation 3 plate reader (BioTek, Winooski, VT, USA), at 570 nm absorption with subtraction of the 690 nm absorption, was used to quantify dissolved Formazan. The control cells were considered to be 100% viable and cell viability of the treated cells were calculated as a percentage of control cell absorbance as shown in Equation (1).

Equation (1). Determination of Cell Viability Using MTT.

(1) $$\mathrm{Cell}\;\mathrm{viability}\;\left(\%\right)=\frac{\mathrm{Absorbance}\;\mathrm{of}\;\mathrm{Folic}\;\mathrm{acid}\;\mathrm{or}\;\mathrm{Selenofolate}-\mathrm{treated}\;\mathrm{cells}\;}{\mathrm{Absorbance}\;\mathrm{of}\;\mathrm{control}\;\mathrm{cells}}\times 100$$

### 2.10. Detection of Intracellular Superoxide Generation with Dihydroethidium

Two hundred thousand MDA-MB-468 and HME50-5E cells were seeded into each well of 24-well flat bottom plates. After 48 h, phenol red-free DMEM high glucose supplemented with FBS was added to each well. Superoxide Dismutase (SOD) from bovine erythrocytes was added to each well (50 U) along with 100 U/well of Catalase from bovine liver dissolved in medium. All cells; control, Folic Acid (100 µM), Selenofolate (100 µM) and selenite (20 μM), were treated for 30 min. Dihydroethidium (DHE) was added at a final concentration of 10 µM/well. Cells were photographed at 30 min and visually assessed using the RFP channel using the EVOS FL Auto Cell Imaging System (ThermoFisher Scientific Carlsbad, CA, USA). The superoxide indicator dihydroethidium, also called hydroethidine, exhibits blue-fluorescence in the cytosol until oxidized, where it intercalates within the cell’s DNA, staining its nucleus a bright fluorescent red. Dihydroethidium was chosen as it expresses superoxide generation throughout the whole cell and our tests were simultaneously measuring superoxide throughout the whole cell at the same time the cells were undergoing oxidative stress when exposed to either Selenofolate or selenite.

### 2.11. MitoTracker^®^ Red and Annexin V Staining for Apoptosis

Cells were seeded at 40,000 cells/well into 48-well, flat-bottom plates and cultured for 5 days prior to commencement of treatment. Media was changed on day 3. On day 5 post seeding, cells were treated with 50, 75 and 100 µM of Folic Acid or Selenofolate and incubated for 3-days, post-treatment. Control cells were treated similarly, but without Folic Acid or Selenofolate. Control cells were treated with 4 µM/well (2 µg Se) as selenite. To induce necrosis, cells were treated with 50 µL of 0.01% Triton X-100 for 30 min or with 200 µL of H_2_O_2_ for 24 h, prior to staining. Single stained controls were treated with Annexin V–488 and the apoptosis inducer Sutent (5 µL of 1 mM solution). Double stained controls cells were treated with the same volume of both dyes. These controls were retained in 2 mL Eppendorf tubes. All treatments and analyses were performed in triplicate.

A 10 mM stock solution of MitoTracker^®^ Red dye was prepared by adding 9.4 μL DMSO to the vial. The working solution of MitoTracker^®^ Red dye at 10 μM, was prepared by adding 1 μL of the 10 mM solution into 1000 μL of DMEM high glucose, phenol red free cell medium. Two microliters of the 10 μM MitoTracker^®^ Red working solution was then added to each well containing 500 μL of medium and stained for 30 min at 37 °C under 5% CO_2_. After incubation, the cells were washed with 500 µL of PBS and trypsinized using 200 µL of 0.025% trypsin-EDTA, centrifuged and collected into sterile 2 mL Eppendorf tubes. Cells were resuspended in 100 µL of 1× Annexin-binding buffer and transferred into 96 well flat-bottom plates. Annexin V dye (4 μL) was added to each well and wrapped in aluminum foil. After a 15 min incubation at 37 °C in 5% CO_2_, 100 µL of 1× Annexin-binding buffer was added to the cells. The plates were immediately placed on ice and results were quantitated by flow cytometry using an Attune™ NxT Flow Cytometer (ThermoFisher Scientific, Carlsbad, CA, USA). Dye detection parameters were set at BL1 (200 nm) and YL1 (336 nm). Forward Scatter voltage was 120 V with Side Scatter at 290 V.

### 2.12. Statistical Analyses

All experimental assays were conducted in triplicate and the data are representative of three independent experiments. The results are expressed as the mean ± standard error (SE). Statistical analyses were performed using MATLAB (2017A) two sample t test across treatments. Differences were considered significant at *p* ≤ 0.05. IC50 was calculated using a two-parameter sigmoidal model. The results of the comparative statistical analyses are summarized in Tables 1 and 2 and the ANOVA results are included in the Supplemental Materials as Table S1.

## 3. Results 

Progress in molecular biology has resulted in both an identification and greater understanding of molecular biomarkers that may have prognostic and predictive value for breast cancer patients. The FRA has been and is again being recognized as a potential therapeutic target for cancer therapy [26], as it has been used for the delivery of radioisotopes, fluorescent molecules for ovarian surgery [16], and for the development of vinblastine conjugates [27]. Patients with breast and other cancers demonstrating overexpression of the FRA receptor protein is associated with a poor clinical prognosis [28]. The overarching goal of newer therapeutic strategies is to discover chemotherapeutic agents that selectively target cancer cell proliferation, thus decreasing bystander effects and systemic toxicity of normal cells. Over-expression of the FRA has been reported in many types of cancer [26] and in this present study, we adopted organic chemistry to covalently couple redox Se with Folic Acid as shown in Equation (1), resulting in the selenium conjugate, Selenofolate (Figure 1 and Figure 2). This conjugate was tested for effectiveness against MDA-MB-468 TNBC cells and results were compared to a control non-cancerous, mammary epithelial cell line (HME50-5E), treated concurrently and similarly with Folic Acid, Selenofolate and selenite.

### 3.1. Determining the Redox Potential of Selenofolate

To determine the redox potential of the synthesized conjugate, Selenofolate and Folic acid, Lucigenin superoxide chemiluminescence detection was applied and results measured using a Turner TD-20e Luminometer. Figure 3 demonstrates the experimental CL results. Over a 5-min CL counting period, the observed reaction between Selenofolate and glutathione (GSH) was dependent upon the concentration of GSH. In these reactions, Lucigenin was reduced by the superoxide anion generated and a measurable chemiluminescent reaction occurred. These experiments confirmed the generation of superoxide from the oxidation of GSH by selenite as shown by Seko et al., [23]. Furthermore, the generation of superoxide from the oxidation of GSH was observed in Selenofolate but not CL cocktail or Folic acid.

### 3.2. FRA Expression in MDA-MB-468 and HME50-5E Cells through All Treatments

**Figure 4:**
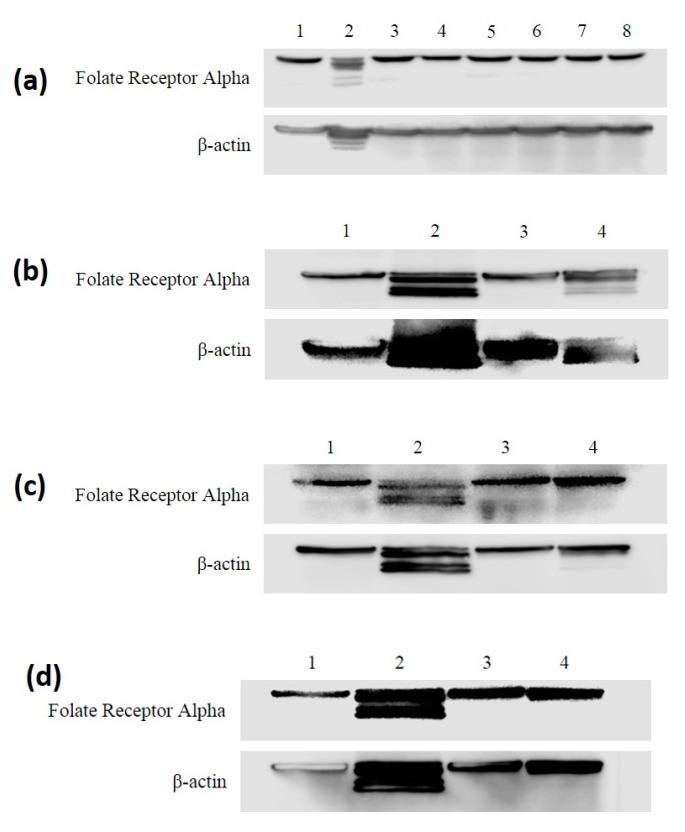
Western blot analysis of the Folate Receptor Alpha in MDA-MB-468 and HME50-5E cells treated with Selenite, Folic Acid, and Selenofolate. Immunoblotting was performed on whole cell lysates with the anti-Folate Receptor Alpha (FRA) or anti β-Actin antibodies followed by horseradish peroxidase conjugated rabbit anti-mouse antibodies and visualized with the SuperSignal™ West Femto Maximum Sensitivity detection system. (**a**) MDA-MB-468 and FRA: Lane 1: control, Lane 2: selenite 4 µM (2 µg Se) treatment, Lane 3: Folic Acid 50 µM treatment, Lane 4: Folic Acid 75 µM treatment, Lane 5: Folic Acid 100 µM treatment, Lane 6: Selenofolate 50 µM (4 µg Se) treatment, Lane 7: Selenofolate 75 µM (6 µg Se) treatment, Lane 8: Selenofolate 100 µM (8 µg Se) treatment. (**b**) HME50-5E and FRA: Lane 1: control, Lane 2: selenite 4 µM (2 µg Se) treatment, Lane 3: Folic Acid 50 µM treatment, Lane 4: Selenofolate 50 µM (4 µg Se) treatment. (**c**) HME50-5E and FRA: Lane 1: control, Lane 2: selenite 4 µM (2 µg Se) treatment, Lane 3: Folic Acid 75 µM treatment, Lane 4: Selenofolate 75 µM (6 µg Se) treatment. (**d**) HME50-5E and FRA: Lane 1: control, Lane 2: selenite 4 µM (2 µg Se) treatment, Lane 3: Folic Acid 100 µM treatment, Lane 4: Selenofolate 100 µM (8 µg Se) treatment.

Western blotting was used to detect the protein expression levels of Folate Receptor Alpha on the surface of MDA-MB-468 and HME50-5E cells (Figure 4). β-actin was used as loading control. A band for the FRA protein was detected at ~38 kDa and β-actin ~42 kDa in control cells, Folic acid and Selenofolate treatments. Selenite show cleaved expression for both FRA and β-actin. One possibility for cleaved FRA expression in selenite treatment is its non-specific toxicity. Cleavage of β-actin by selenite treatment was found in both cell lines tested. Observation of this phenomenon may hold biological and therapeutic implications involving cell–cell interactions, cell proliferation and/or migration and metastasis [29]. In a reported Phase I clinical trial, no major toxicity was identified when sodium selenite, at a dosage of 10.2 mg/m^2^, was administered to cancer patients [29]. Since this observation did not affect the overall Selenofolate results of our project, it was not pursued. While outside the scope of this project, more study is required to address this interesting phenomenon. 

### 3.3. Analysis of Dosage/Time Dependent Cell Viability by Trypan Blue Exclusion

**Figure 5:**
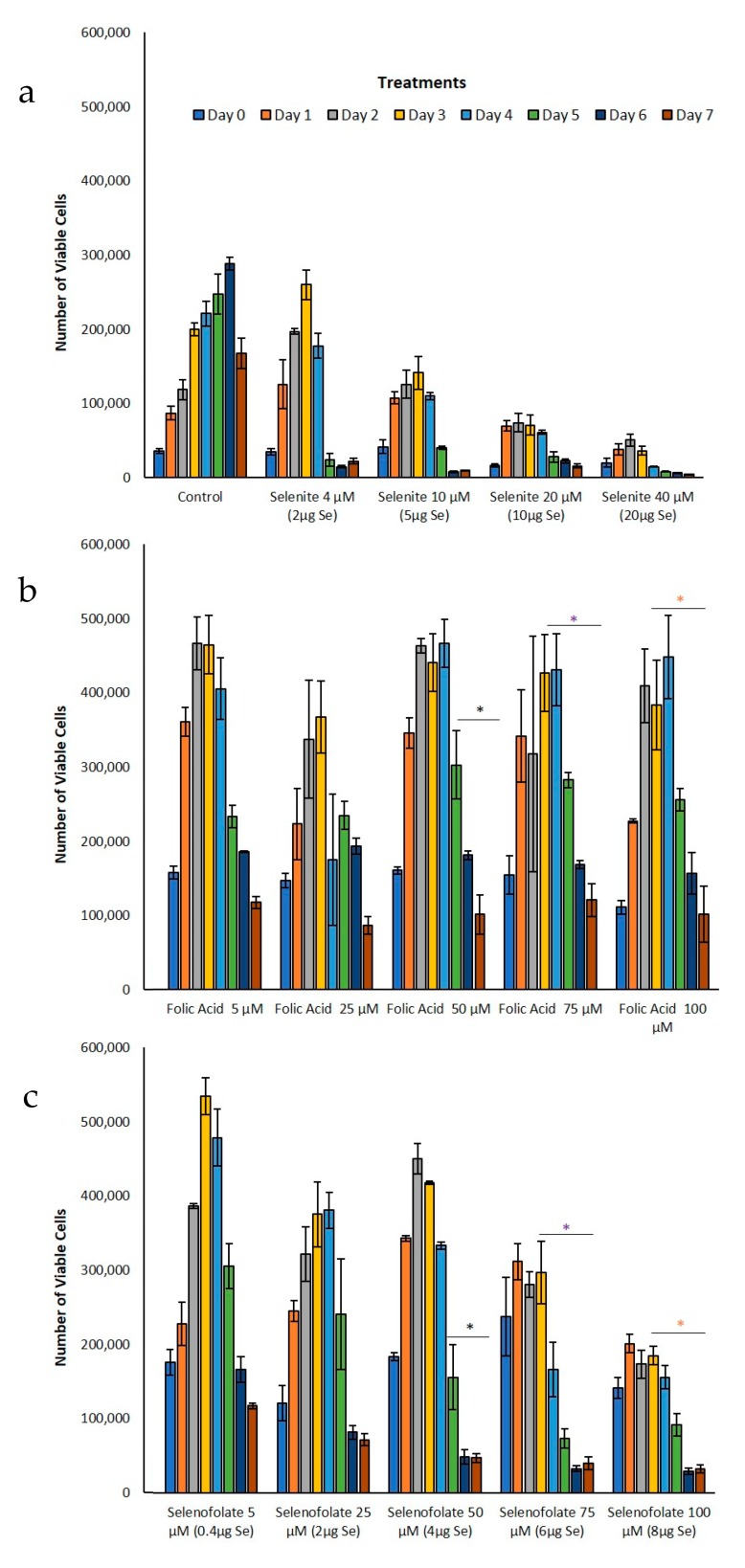
Analysis of treatment cytotoxicity in MDA-MB-468 cells. Cytotoxic effects of (**a**) control and selenite, (**b**) Folic Acid, (**c**) Selenofolate treatments against MDA-MB-468 cells was determined as a function of percent cell viability. Forty-thousand cells were seeded and treated as shown (Day 0 = start of treatment) with 5 μM, 25 μM, 50 μM, 75 μM and 100 μM folate and Selenofolate with Se equivalents of 0.4 μg, 2 μg, 4 μg, 6 μg, and 8 μg respectively. Viable cells were counted and analyzed by Trypan Blue exclusion every 24 h for 7 days. The data are expressed as mean ± SE (*n* = 3). Treatment results were compared using two sample t-tests and considered statistically significant if *p* ≤ 0.05 (indicated by *). Treatments that were statistically significant, *p* ≤ 0.05 are summarized in Table 1.

**Figure 6:**
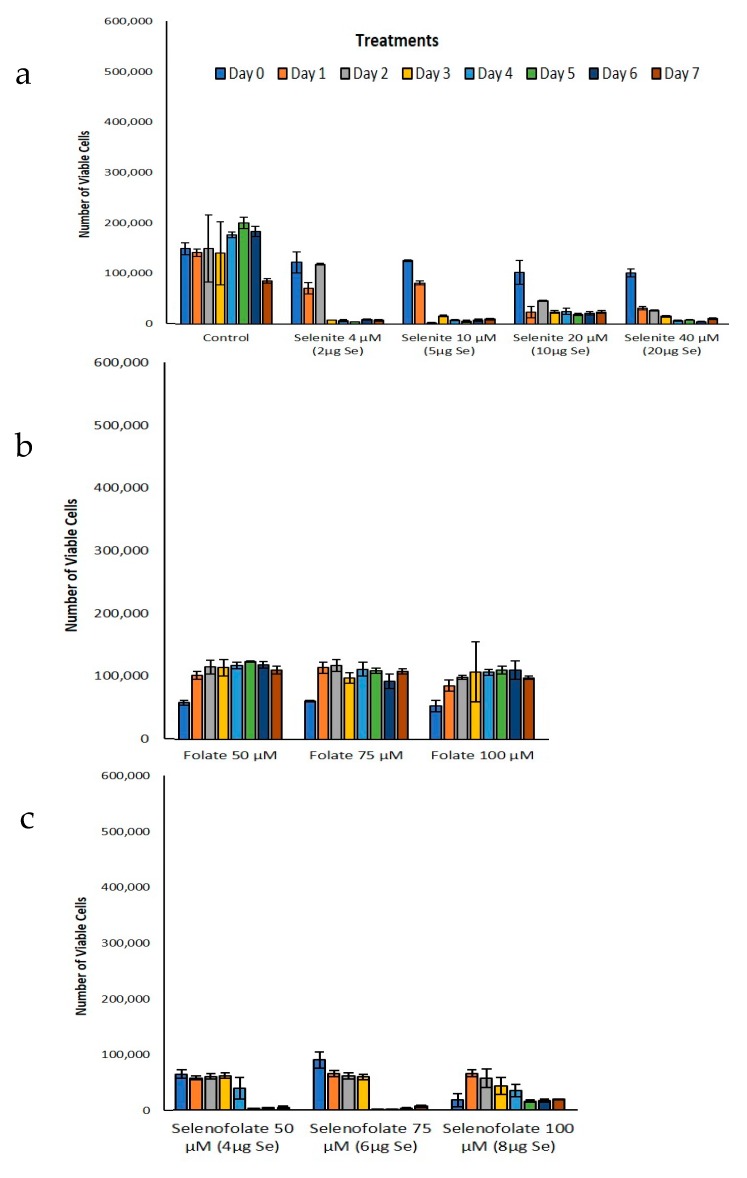
Analysis of treatment cytotoxicity in HME50-5E cells. Cytotoxic effects of (**a**) control and selenite, (**b**) Folic Acid, (**c**) Selenofolate treatments against HME50-5E cells was determined as a function of percent cell viability. Forty-thousand cells were seeded and treated as shown (Day 0 = start of treatment) with 5 μM, 25 μM, 50 μM, 75 μM and 100 μM Folic acid and Selenofolate with Se equivalents of 0.4 μg, 2 μg, 4 μg, 6 μg, and 8 μg respectively. Viable cells were counted and analyzed by Trypan Blue exclusion every 24 h for 7 days. The data are expressed as mean ± SE (*n* = 3). Statistical treatments were compared using two sample t-tests. Treatments that were statistically significant, *p* ≤ 0.05 are summarized in Table 2. Note: Many HME50-5E cells remained adherent to the plate and thus could not be accurately analyzed.

Following the optimization of cell growth, MDA-MB-468 cells and HME50-5E cells were treated with Folic Acid, Selenofolate and selenite. The dose response results of control cell growth and the three treatments as measured by Trypan Blue exclusion and cell viability for TNBC cells are shown in Figure 5 and HME50-5E cells in Figure 6. These experimental treatments show that selenite and Selenofolate induced a general loss of cell viability over increasing selenium dosage and time as measured against control and Folic Acid treated cells. These results are consistent with the ability of Selenofolate to generate superoxide in the in vitro CL assay (as shown in Figure 3). 

Selenofolate 100 µM had cytotoxic effects on MDA-MB-468 and HME50-5E cells. Overall treatment of Selenofolate at 5 µM and 25 µM dose did not cause significant reduction in MDA-MB-468 cell viability. This may be due to selenium reverting to its nutritional role in support of Glutathione peroxidase and other selenium proteins. In total, 2, 5, 10 and 20 µg of Se (as selenite) per well was cytotoxic to both cell types tested. Sodium selenite an inorganic chemical form of selenium is toxic and inhibits cell proliferation by apoptosis not only in human and mouse breast cancer cell lines but also in human colon carcinoma cells, human ovarian cancer, human prostate cancer cells in vitro [30].

Trypan Blue exclusion experiments were not conducted with HME50-5E at 5 µM and 25 µM Selenofolate. In part, successful analysis was difficult due to the strong adherence properties of the HME50-5E cells, causing experimental bias. Initially, 200 µL of 0.025% trypsin-EDTA was added and incubated for 10–15 min but when viewed under a microscope not all the cells completely detached. So, another 200 µL of 0.025% trypsin-EDTA was added to cells to try to collect the whole population. Despite incubating with double the amount (400 µL) of trypsin, a substantial number of cells remained. This inability to adequately remove all HME50-5E cells from the plate resulted in a high percentage loss of cells not included in the Trypan Blue assay, poorly reflecting HME’s viability.

### 3.4. Analysis of Cell Metabolism/Viability by MTT

**Figure 7:**
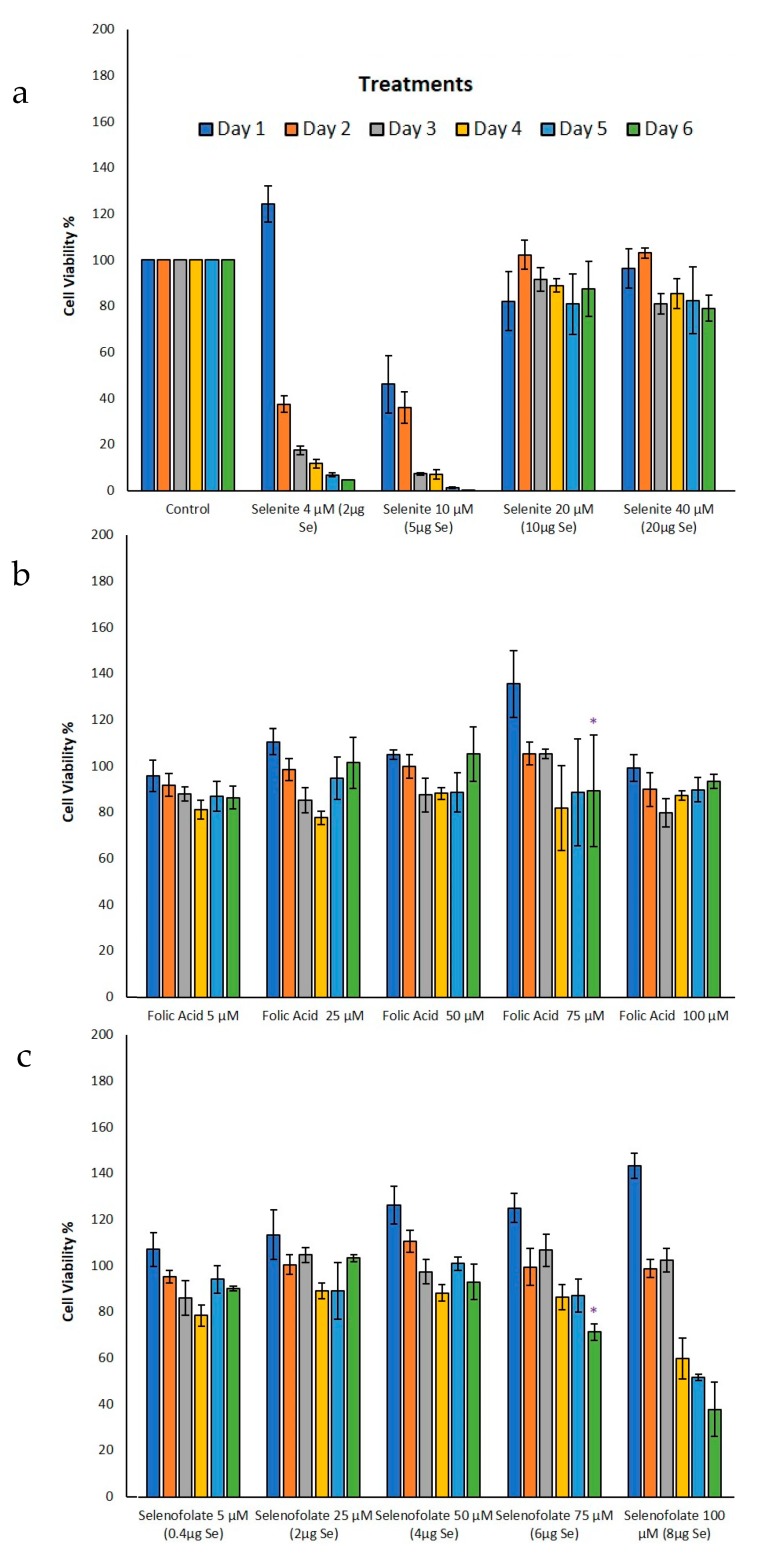
Analysis of metabolic activity as a function of percent cell viability during treatment of MDA-MB-468 cells. Effects on metabolic activity in (**a**) control and selenite, (**b**) Folic Acid, (**c**) Selenofolate treatments against MDA-MB-468 cells as determined by the MTT assay. Forty-thousand cells were treated as shown (Day 0 = start of treatment) with 5 μM, 25 μM, 50 μM, 75 μM and 100 μM Folic acid and Selenofolate with Se equivalents of 0.4 μg, 2 μg, 4 μg, 6 μg, and 8 μg respectively. The data represent the mean ± SE (*n* = 3). Treatments were compared using two sample t-tests and treatments that were statistically significant at *p* ≤ 0.05 (indicated by *) are summarized in Table 1.

**Figure 8:**
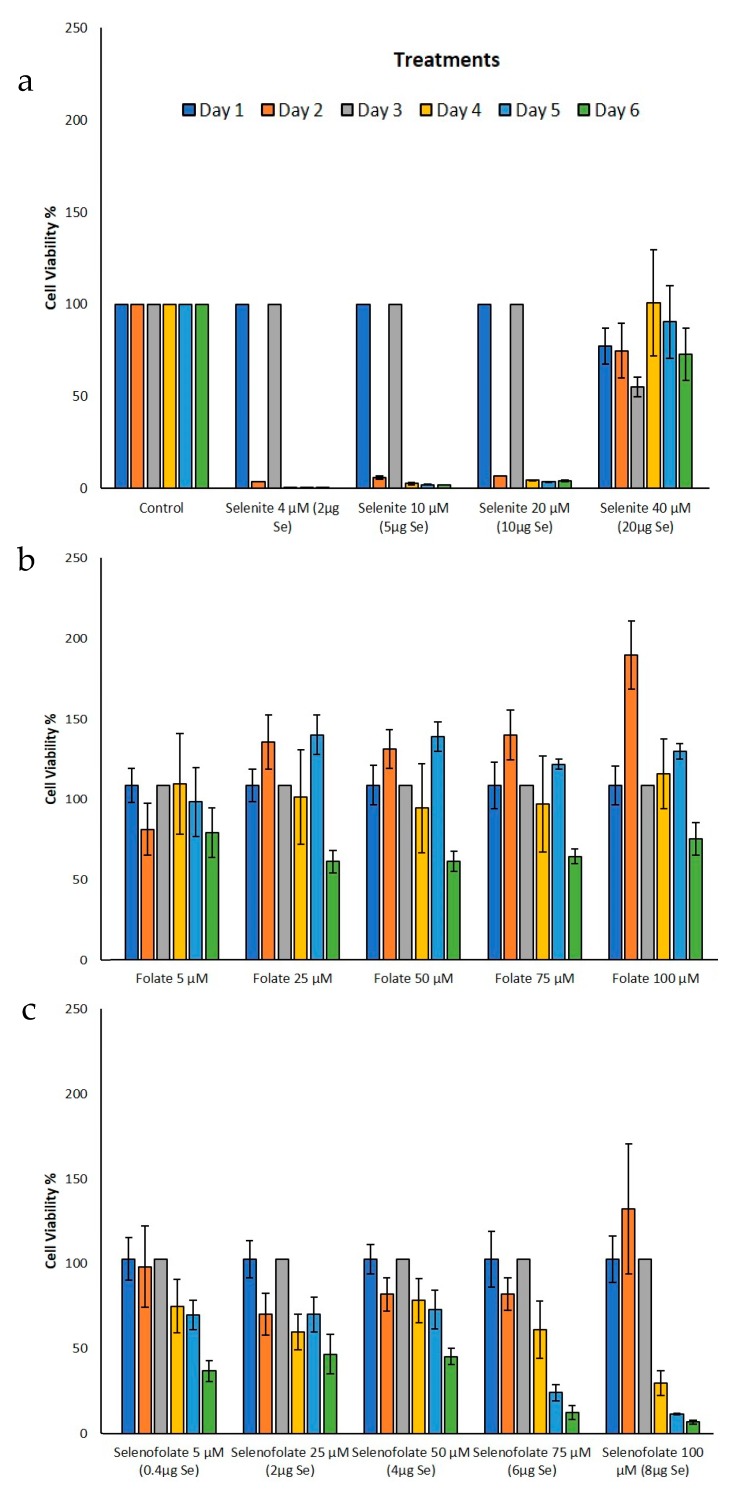
Analysis of metabolic activity as a function of percent cell viability during treatment of HME50-5E cells. Metabolic activity of (**a**) control and selenite, (**b**) Folic acid and (**c**) Selenofolate treated HME50-5E cells was determined by the MTT Assay. Forty-thousand cells were treated on day 5 post seeding (day 0 treatment) with 5 μM, 25 μM, 50 μM, 75 μM and 100 μM Folic acid and Selenofolate with Se equivalents of 0.4 μg, 2 μg, 4 μg, 6 μg, and 8 μg, respectively. The cell viabilities are shown as Means ± SE (*n* = 3). Treatments were compared using two sample t test and treatments that were statistically significant at *p* ≤ 0.05 are summarized in Table 2. Note: HME50-5E cells were very adherent, highly metabolic and surpassed the detection capabilities of the colorimetric readings (see text and Supplementary Materials).

To gain insight into the Trypan Blue exclusion results, the MTT assay (3-[4,5-dimethylthioazol-2-yl]-2-5-diphenyltetrazolium bromide) was performed to assess cell viability through metabolic activity. For this analysis, viable cells cleave a tetrazolium salt ring to form the insoluble Formazan. This method would also elucidate cell viability status in the HME50-5E cells when treated with Selenofolate. The results for the cell lines tested are illustrated in Figure 7 (MDA-MB-468) and Figure 8 (HME50-5E). Se as selenite was toxic to cells at all concentrations. Folic acid treatments alone showed high cell viability for all doses and time periods, as Folic acid is non-toxic at these concentrations and serves as a nutrient for cancer cells. MDA-MB-468 cell viability ranged from 97% to 102% for 100 μM (8 μg Se) Selenofolate and 50 μM (4 μg Se) Selenofolate, (Figure 7). Due to this nutritive nature and as expected, days 1–3 did not demonstrate significant differences in cell viability between Folic acid and Selenofolate in MDA-MB-468 cells. However, starting at day 4 for 100 μM Selenofolate (8 μg Se) and day 6 for 75 μM Selenofolate (6 μg Se), significant differences in cell metabolism/viability were observed between Folic acid and Selenofolate treatments.

MTT incubation in the HME50-5E cells yielded an interesting result. When the MTT solubilization media for Formazan was added to HME50-5E cells, the Formazan salt could not be dissolved completely because of the adherence properties of the cells (Figure S2). Also, the intensity of Formazan color was so dark that the plate reader displayed ‘OVERFLOW’ for results on day 1 and day 3 of treatments indicating that the metabolic activity was so high, it saturated the limits of detection for the instrumentation. This observation coupled with the confluent, adherent cultures, suggested that the vast majority of the population was metabolically healthy following Se treatments. Control HME50-5E cell viability was therefore set at 100% viability for days 1 and 3 treatments. Moreover, no significant differences were observed on day 3 for Folic acid or Selenofolate treatments. Ip [31] suggested that normal, early transformed and late stage preneoplastic cells may demonstrate differential responses to selenium intervention with respect to molecular pathways involving cell cycle proteins and apoptotic proteins. The photomicrographs reflect this result (shown in Figures S1 and S2). While morphological changes due to selenite treatment was observed in both cell types, treatment with Selenofolate was much more apparent in the MDA-MB-468 cells with the HME 50-5E cells showing little effect.

### 3.5. Generation of Intracellular Superoxide in Treated Cells

**Figure 9:**
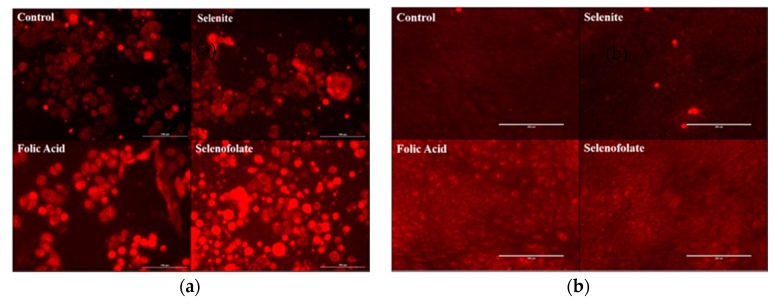
Photomicrographs of intracellular superoxide generation by Dihydroethidium (DHE) red florescence. (**a**) MDA-MB-468 Cells (left panels) with scale bar at 100 µm. (**b**) HME50-5E cells (right panels) with scale bar at 200 µm. Results were photographed after 30 min of treatment with untreated control, 20 µM selenite (10 µg Se), 100 µM Folic Acid and 100 µM Selenofolate (8 µg Se).

After determining the chemiluminescence activity of Folic acid and Selenofolate at different GSH concentrations, coupled with the interesting and frustrating results from the Trypan Blue and MTT experiments (specifically with regards to visual observation versus in vitro assay results for the HME50-5E cells), it was important to determine if Folic acid and/or Selenofolate would generate superoxide in situ. The photographs taken on an EVOS FL Auto Cell Imaging System in Figure 9a (TNBC) and Figure 9b (HME50-5E) reveal the intracellular production of superoxide by Selenofolate and selenite, but not Folic acid, analyzed using the DHE assay. Over time, Selenofolate and selenite treated cells show gross cell morphological changes in the MDA-MB-468 cells with increasing selenium concentration; observed as cell shrinkage, swelling, and cell membrane disruption. Very low concentrations of Selenofolate as well as very low selenite concentrations <1 ug Se, were not expected to show toxicity to cells as the selenium reverts to its metabolic role in the support of Glutathione peroxidase and the synthesis other selenium containing proteins [31].

Similar results have been reported from prior studies in our laboratory comparing the cytotoxicity of selenite, selenate, selenocystine and selenomethionine towards the human mammary tumor cell lines HTB123/DU4475 in vitro [32]. In contrast to the TNBC cell line, Selenofolate did not induce superoxide production in HME50-5E cells (Figure 9b). This difference between TNBC cells’ and the HME50-5E cells’ resistance to redox selenium suggests a biochemical variance that can provide a basis for more selective killing of cancer cells with reduced toxicity to normal cells. The results are consistent with cancer cells being known to overexpress FRA relative to normal cells (with kidney cells as the one exception). 

### 3.6. Analysis of Apoptosis-Induced Cell Death after Treatment with Selenofolate

**Figure 10:**
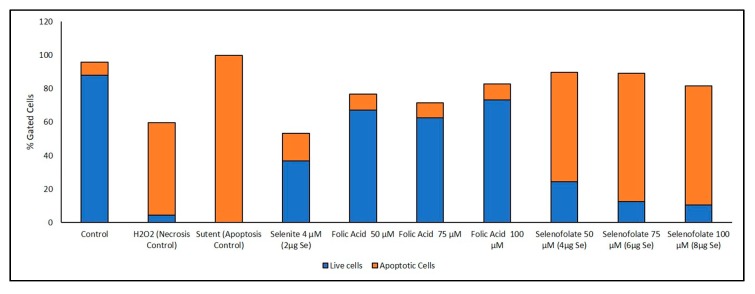
Graphical representation (as percent of cells) of apoptotic events following Selenofolate treatment. Percent distribution of MDA-MB-468 apoptotic cells after treatment with H_2_O_2_ (necrosis inducer), Sutent (apoptosis inducer), selenite (positive control), Folic Acid and Selenofolate treatments, or untreated. Data are expressed as the mean (*n* = 3) and is based upon 100% of cells gated.

**Figure 11:**
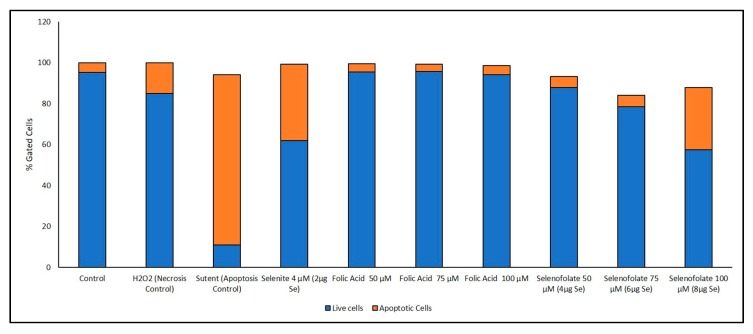
Graphical representation (as percent cells) of apoptotic events following Selenofolate treatment. Percent distribution of HME50-5E apoptotic cells after treatment with H_2_O_2_ (necrosis inducer), Sutent (apoptosis inducer), selenite (positive control), Folic Acid and Selenofolate treatments, or untreated. Data are expressed as the ean (*n* = 3) and is based upon 100% of cells gated.

Previous studies have established that selenium induces apoptosis by the intrinsic (mitochondrial-mediated) pathway [33,34,35,36,37]. Furthermore, apoptosis or programmed cell death (PCD) is associated with morphological, biochemical and molecular changes occurring in the cell [38]. In order to confirm that Selenofolate treatments also induce apoptosis, an Annexin V assay was performed. Both Selenofolate and selenite treated cells showed prominent morphological changes, characteristic of apoptosis as evidenced by photomicrographs (Figures S3 and S4). Due to different sensitivities identified at day 3 for all cell lines tested in both Trypan Blue and MTT assays, cells were incubated with three different concentrations of Selenofolate to determine whether apoptosis would be induced. Due to its established apoptotic activity, Sutent (Pfizer) was used as the positive control for apoptosis induction in TNBC [39] and HME50-5E cells. H_2_O_2_ was used as the positive control for necrosis induced cell death. Flow data for apoptosis and live cells were determined based on ratios of the presence of Annexin V and intensity of MitoTracker Red CMXRos in the controls for cell death and the signal intensity for both dyes associated with live untreated cells. The percentages of these ratios are graphically illustrated in Figure 10 and Figure 11. Raw flow cytometry data are included in the Supplementary Materials as Figures S5 and S6.

Interestingly, Sutent demonstrated even greater potency in killing the normal cells (Figure 11). To our knowledge, this is the first report of toxicity of Sutent on normal cells. This suggests important consequences for clinical success of Sutent with respect to side effects. Further study is needed to investigate this observation. Control and Folic acid treated cells also showed apoptosis (Figure 10 and Figure 11). However, the effect was greater in the TNBC cells. One possibility for this phenomenon is that the rapidly dividing cells became overconfluent in the limited well space, causing cell death. Increased cell viability and cell proliferation was observed in Folic acid alone treatments in both Trypan Blue exclusion and MTT (Figure 5 and Figure 7, respectively). Apoptotic cell populations in the vehicle control of prior studies of candidate anticancer therapies have also reported this phenomenon in their control groups [36,40].

A loss of mitochondrial membrane potential (ΔψM) indicates a reduced cell viability due to perturbation of the proton pumps across the inner membrane during the process of electron transport and oxidative phosphorylation driving the conversion of ADP to ATP [41]. Therefore, MDA-MB-468 cells and HME50-5E cells were stained with the live cell MitoTracker Red CMXRos (instead of Propidium Iodide), the accumulation of which is dependent upon the ΔψM. In this study the ΔψM was measured by flow cytometer and the results showed a Selenofolate dose-dependent decrease in signal intensity and a concurrent dose-dependent increase in the percent apoptotic MDA-MB-468 cells. In contrast, there was no change in the ΔψM (UL quadrant) of control vs. Selenofolate treated HME50-5E cells (Figure 11 and Figure S5), indicating live, viable cells. This validates the intense Formazan color observed from MTT assay assessing cellular metabolic activity (Figure S1). Hence, HME50-5E cells do not undergo apoptosis following Selenofolate treatment. These observations again imply the role of selenium-induced oxidative stress/glutathione triggered-apoptosis associated with both a decrease in mitochondrial GSH levels and an increased generation of superoxide as well as ΔψM in the cancer cells. Studies have reported that one of the most affected pathways of Se compounds in cancer therapy is the modulation of mitochondrial functions regulating apoptosis [42]. Selenite also caused necrosis in MDA-MB-468 cells, while few events were detected in HME50-5E cells. Selenite is known to induce necrosis in other breast cancer cells [31].

## 4. Discussion

The experimental results from this study corroborate those studies that show the cytotoxic effects of selenocompounds that form selenides (RSe–) in different cultured malignant cancers [43]. The MTT assay and Annexin flow cytometry results in Figure 7 and Figure 10 substantiates the loss of cell viability most likely from the induction of apoptosis, the selenium oxidative redox chemistry targeting reduced GSH and other thiols, the cysteines of the mitochondrial membrane, and its membrane potential [34]. The selenium literature broadly supports the experimental observation that cancer cells are usually more susceptible to selenium apoptosis and cell death than their corresponding normal progenitor cells. The HME50-5E cells were found somewhat difficult to test because of their extreme substrate adherence. These cells had to be treated with extra lyase for release, but nevertheless they were found to be less susceptible to Selenofolate and selenite toxicity than the MDA-MB-468 cells as shown in Figure 8 and Figure 11. This also correlated with the expression of the FRA on these cell lines as shown in Figure 4, the generation of intracellular superoxide as measured by the DHE fluorescence (Figure 9) and the general lack of morphological changes in the morphology of the cells from control or selenium treated HME50-5E cells (Figure S3). It should also be noted that the same experiments were performed on MDA-MB-231 cells, and although slightly more resistant than their 468 TNBC counterparts, the data were very similar.

As discussed previously, the FRA is upregulated and over-expressed on many cancer cells lines [12,44,45]. FRA is thus a potential major target for the clinical treatment of TNBC with redox selenium Folic Acid conjugates or a redox selenium FRA specific mAb. Studies that report that the Folic Acid conjugated radioisotopes are directed to only cancer and normal kidney cells [46], that folate-fluorescein conjugates can be internalized by ovarian cancers [15] and that Vintafolide, folate-vinblastine conjugates were not effective in a Phase 3 clinical trial [19] opens up the possibility that small redox conjugates like Selenofolate, that does not require disassociation from Folic Acid may be a significant treatment modality for many cancers frequently detected and diagnosed at Stage 4.

The present study demonstrated the synthesis of Selenofolate from Folic Acid, and that Selenofolate as with selenite, both generated superoxide, which reduced MDA-MB-468 cell viability and proliferation in vitro as a function of dose and time over Folic Acid alone. The decreased cell viability and proliferation of the MDA-MB-468 cells by Selenofolate and selenite were likely intrinsic apoptosis initially induced by intracellular superoxide generation. These conclusions are drawn from the data assessment of the identification of the FRA protein using Western blot analysis, cell morphology, cell viability subjected to Trypan Blue staining, the MTT assay, the visual assessment of internally generated superoxide using DHE, and the detection of apoptosis by flow cytometry.

The major concern is the potential toxicity of Selenofolate to cells other than cancer cells in an in vivo trial. Although Folic Acid receptors are expressed at very low levels in normal cells, Weitman et al. [12] reported that normal renal epithelium cells express high amounts of the FRA. Due to its small molecular weight, Selenofolate (MW = 573 g/mol) may be easily reabsorbed in the proximal tubules of the kidney after excretion. Thus, Selenofolate may potentially accumulate in the kidneys and possibly result in organ damage. However, Leamon and Reddy [47] found that the FR–drug conjugate EC72 showed effective anticancer characteristics in mice. No harm was caused to normal tissues, including the FR-positive kidney cells. Radioisotope labeled Folic Acid and imaging in mice revealed that only cancer cells and kidney cells accumulate radio-folate. No other cells demonstrated isotopic imaging. This suggests that Selenofolate may not be cytotoxic to normal cells as Selenofolate and selenite were both almost non-toxic to the non-cancerous breast epithelial cell line, HME50-5E by morphological, superoxide generation and apoptosis analyses. Furthermore, since the Selenofolate treated HME50-5E cells were the most difficult to dissociate from the substratum, the possibility exists that the selenium–Folic Acid conjugate somehow enhanced the adherent properties of the epithelial cells. However, this speculation needs to be researched. 

As noted, Folic Acid has been employed to target cancer with radioisotopes, fluorescent molecules and chemotherapeutic drugs. The failure of the Phase 3 trial of Vintafolide underscores the problem of large chemotherapeutic molecules, in this example Vinblastine, (MW 811 g/mol) conjugated to a small targeting molecule, Folic Acid and its receptor protein, the FRA. From our perspective, failure of these large chemotherapeutic molecular conjugates likely resides in reduced cellular assimilation due to size restrictions, reduced receptor affinity and/or that the carcinostatic drug may not undergo hydrolysis from its conjugate, negating chemotherapeutic cytostatic activity if internalized. Covalent redox selenides pose no such obstacles. The selenium modification is very small in comparison to fluorescent tags that are absorbed and large chemotherapeutic drugs that do not work well, as no dissociation of the redox selenium from its conjugate is required. The redox selenium is concentrated by its covalent targeting molecule and goes right to work generating internal cellular oxidative stress in constitutively proliferating cells, i.e., cancer. This is true for Selenofolate as well as any other targeting mAb [21] or other protein as shown for transferrin [20].

## 5. Conclusions

In conclusion, this in vitro study demonstrated that the FRA protein expression from both cell lines was similar and interestingly, we observed cleaved β-actin from Selenofolate, and selenite treated cells. With β-actin’s 3 cystine disulfides, reduction of these disulfides may also be a consequence of selenium’s internal redox chemistry. Selenofolate was internalized, most likely through the FRA. A time and dose decrease in cell viability and proliferation was observed for the MDA-468 cells, but not the breast epithelial HME50-5E (as confirmed by the redox active selenium assay). The results from the Trypan Blue and MTT assays of viability at varied Selenofolate selenium concentrations demonstrated significant (*p* ≤ 0.05) loss of MDA-MB-468 viability compared to control assays with less effects on the HME50-5E cell viability. Annexin V assays demonstrated that cell death was due to apoptosis in the MDA-MB-468 cells with significantly less effect in the HME50-5E cells. Since sodium selenite has long to be known to target and cause mitochondrial swelling [48], apoptosis was likely induced intrinsically. The DHE assay demonstrated that induced superoxide generation was intracellular in the MDA-468 cells but again, no comparable effects were observed in the HME50-5E cells.

Collectively, our experimental results are interpreted as a possible new option of a small molecular weight drug delivery system of redox selenium by Folic Acid in triple negative breast cancer cell treatments where FRA may be over-expressed. While the leap from in vitro cancer cell experimentation to clinical treatments is long and risky (e.g., Vintafolide), since the FRA has been established as being over-expressed on many cancers, Selenofolate would seem to offer a promising alternative option for treating TNBC and other FRA cancers.

## Figures and Tables

**Table 1:** Statistical analyses of the effects of Folic acid and Selenofolate treatments on MDA-MB-468 cells.

Number of Days	Experiment	Treatments	*p* Value	Significance
**Day 3**	Trypan Blue (Figure 5b,c)	Folic acid 100 µM vs. Selenofolate 100 µM	0.015	*
**Day 4**	Trypan Blue (Figure 5b,c)	Folic acid 50 µM vs. Selenofolate 50 µM	0.008	**
		Folic acid 75 µM vs. Selenofolate 75 µM	0.006	**
		Folic acid 100 µM vs. Selenofolate 100 µM	0.004	**
**Day 5**	Trypan Blue (Figure 5a–c)	Control vs. Selenofolate 75 µM	0.020	*
		Control vs. Selenofolate 100 µM	0.035	*
		Folic acid 50 µM vs. Selenofolate 50 µM	0.041	*
		Folic acid 75 µM vs. Selenofolate 75 µM	0.001	***
		Folic acid 100 µM vs. Selenofolate 100 µM	0.001	***
**Day 3**	MTT (Figure 7b,c)	Folic acid 75 µM vs. Selenofolate 75 µM	0.024	*
**Day 4**	MTT (Figure 7a,c)	Control vs. Selenofolate 100 µM	0.022	*
**Day 3**	Annexin V (Early/Late Apoptosis) (Figure 10)	Folic acid 50 µM vs. Selenofolate 50 µM (early)	0.001	***
		Folic acid 75 µM vs. Selenofolate 75 µM (early)	0.001	***
		Folic acid 100 µM vs. Selenofolate 100 µM (early)	0.002	**
		Control vs. Selenofolate 50 µM (early)	0.001	***
		Control vs. Selenofolate 75 µM (early)	0.002	**
		Control vs. Selenofolate 100 µM (early)	0.004	**
		Folic acid 50 µM vs. Selenofolate 50 µM (late)	0.004	**
		Folic acid 75 µM vs. Selenofolate 75 µM (late)	0.001	***
		Folic acid 100 µM vs. Selenofolate 100 µM (late)	0.001	***
		Control vs. Selenofolate 50 µM (late)	0.003	**
		Control vs. Selenofolate 75 µM (late)	0.001	***
		Control vs. Selenofolate 100 µM (late)	0.001	***

*p* ≤ 0.05 (*), *p* ≤ 0.01 (**), and *p* ≤ 0.001 (***), 50 µM = 2 µg Se, 75 µM = 6 µg Se, 100 µM = 8 µg Se.

**Table 2:** Statistical analyses of the effects of Folic acid and Selenofolate treatments on HME50-5E cells.

Number of Days	Experiment	Treatments	*p* Value	Significance
**Day 3**	Trypan Blue (Figure 6b,c)	Folic acid 50 µM vs. Selenofolate 50 µM	0.004	**
		Folic acid 75 µM vs. Selenofolate 75 µM	0.002	**
**Day 4**	Trypan Blue (Figure 6b,c)	Folic acid 50 µM vs. Selenofolate 50 µM	0.002	**
		Folic acid 75 µM vs. Selenofolate 75 µM	0.001	***
		Folic acid 100 µM vs. Selenofolate 100 µM	0.001	***
**Day 4**	MTT (Figure 8b,c)	Folic acid 100 µM vs. Selenofolate 100 µM	0.010	*
**Day 5**	MTT (Figure 8b,c)	Folic acid 50 µM vs. Selenofolate 50 µM	0.007	**
		Folic acid 75 µM vs. Selenofolate 75 µM	0.001	***
		Folic acid 100 µM vs. Selenofolate 100 µM	0.001	***

*p* ≤ 0.05 (*), *p* ≤ 0.01 (**), and *p* ≤ 0.001 (***), 50 µM = 2 µg Se, 75 µM = 6 µg Se, 100 µM = 8 µg Se.

## Supplementary Materials

The following are available online at https://www.mdpi.com/2076-3921/9/2/138/s1, Table S1. Summary ANOVA Results for Selenofolate Treatments, Figure S1. Synthesis of selenocyanethanol and Selenofolate, Figure S2. Image of MTT plate on day 3 post-treatment demonstrating presence of insoluble formazan salt, Figure S3. Photomicrographs of the morphological changes observed in MDA-MB-468 cells, Figure S4. Photomicrographs of the morphological changes observed in HME50-5E cells, Figure S5. Folate, Selenofolate and Selenite induced apoptosis in MDA-MB-468 Cells, Figure S6. Folate and Selenofolate did not induce apoptosis in HME50-5E cells.
