# Supplementary material for: Investigating the Potential of Conjugated Selenium Redox Folic Acid as a Treatment for Triple Negative Breast Cancer
Source: Antioxidants (Basel). 2020 Feb 5;9(2):138. doi: 10.3390/antiox9020138 (PMC7071027; doi:10.3390/antiox9020138)
Supplement: Supplementary file 1 [file antioxidants-09-00138-s001.pdf]

antioxidants

Supplementary Materials

Table S1. Summary ANOVA Results for Selenofolate Treatments.

Experiment name	Treatment	Treatment Day	F-value	p-value
Trypan Blue	Folic Acid and Selenofolate	Day 3	F(4,10)= 19.32	0.0001
Trypan Blue	Folic Acid and Selenofolate	Day 4	F(4,10)= 27.02	2.43E-05
Trypan Blue	Folic Acid and Selenofolate	Day 5	F(4,10)= 5.51	0.0131
Trypan Blue	Folic Acid and Selenofolate	Day 6	F(4,10)= 31.8	1.16E-05
Trypan Blue	Folic Acid and Selenofolate	Day 7	F(4,10)= 26.54	2.63E-05
**Experiment name**	**Treatment**	**Treatment Day**	**F-value**	**p-value**
MTT	Folic Acid and Selenofolate	Day 3	F(5,12)= 1.21	0.3612
MTT	Folic Acid and Selenofolate	Day 4	F(5,12)= 4.86	0.0116
MTT	Folic Acid and Selenofolate	Day 5	F(5,12)= 6.43	0.004
MTT	Folic Acid and Selenofolate	Day 6	F(5,12)= 15.56	6.96E-05

*Note: There was an extra treatment of 1 uM in MTT assay.

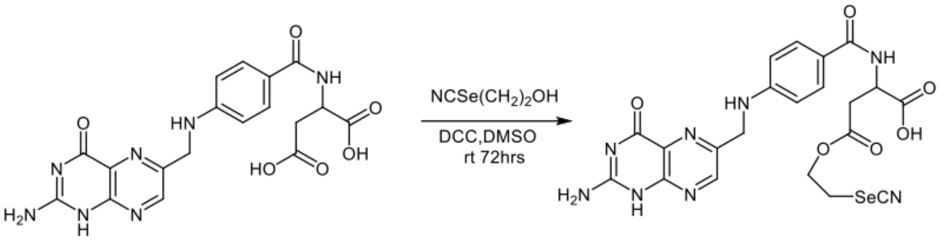

Figure S1. Synthesis of selenocyanethanol and selenofolate.

A substantial proportion of MDA-MB-468 cells in the upper right (UR) and lower right (LR) quadrant (as shown in **Figure S4**), correspond to early and late apoptosis respectively. The morphological phenotypes (**Figure S2**) of these apoptotic cells are strongly correlated with flow analysis. In contrast, considerable proportions of HME50-5E cells were found in the upper left (UL) quadrant (as shown in **Figure S5**), corresponding to living cells and reflective of the lack of morphological changes observed in **Figure S3**.

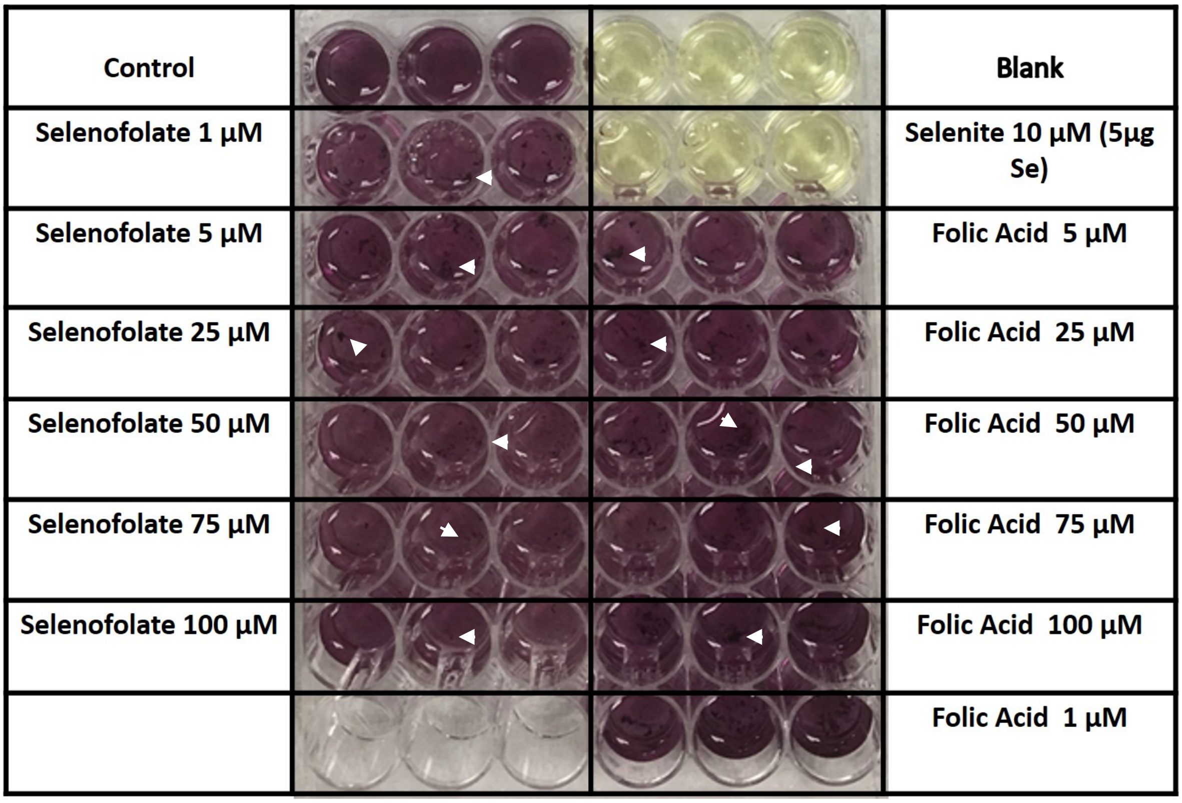

Figure S2. Image of MTT plate on day 3 post-treatment demonstrating presence of insoluble Formazan salt. The dark purple precipitates can be observed by eye (white arrowheads) and the intensity of the color generated by metabolic conversion to Formazan was outside the colormetric range of detection for the plate reader and registered as OVERFLOW. For this assay, the darker the color, the more metabolically active the cells.

To visualize morphological toxicity MDA-MB-468 and HME50- 5E cells were treated with Selenofolate, Folic Acid and selenite as Se. Images were taken under a light microscope at 20X magnification and results are shown in **Figure S2** (MDA-MB-468) and **Figure S3** (HME50-5E). MDA-MB-468 and HME50-5E cells are all adherent epithelial cells. With adherent cells, detachment from the substratum as well as morphological change is indicative of apoptosis [49]. Selanofolate and selenite treated TNBC cells show gross cell morphological changes, with both cell swelling and shrinkage, and cell membrane disruption ultimately detachment from the cell culture substratum. Additionally, the morphological change (from the grape-like structure in MDA-MB-468 cells) became more remarkable with increased treatment exposure. Similar morphological change in cancer cells treated with other Se compounds have been reported in other in vitro studies [31]. From a therapeutic point of view, the clinical relevance of this observation is that Selenofolate can be effective in suppressing tumor growth if targeting and endocytosis is optimized. While selenite demonstrated cytotoxic effects on the HME50-5E cells, Selenofolate did not show significant effects. Interestingly, Folic Acid treatment alone, showed morphological changes from cuboidal, cobblestone-like to fibroblast-like structure in HME50-5E cells (**Figure S3**).

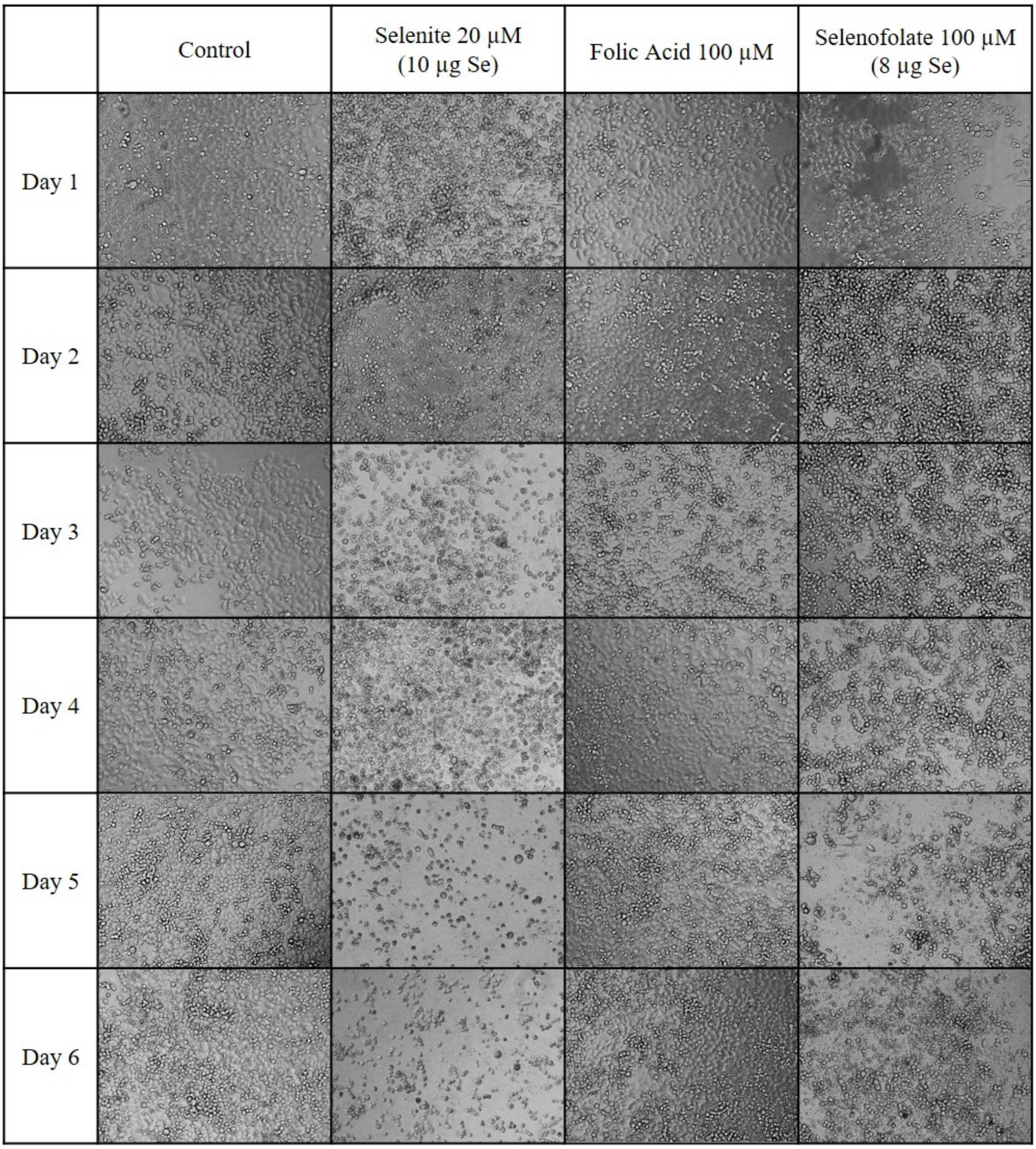

Figure S3. Photomicrographs of the morphological changes observed in MDA-MB-468 cells. Cells were untreated (PBS Control) or treated with 20 μM Selenite (10 μg Se), 100 μM Folic Acid and 100 μM Selenofolate (8 μg Se) over 6 days. Treatment of the MDA-MB-468 cells with 20 μM Selenite and 100 μM Selenofolate revealed morphological changes indicative of membrane disruption and decreased cell viability in comparison to the 100 μM Folic Acid. Representative fields of view of MDA-MB-468 cells. Cells were photographed under phase contrast conditions at 20X magnification.

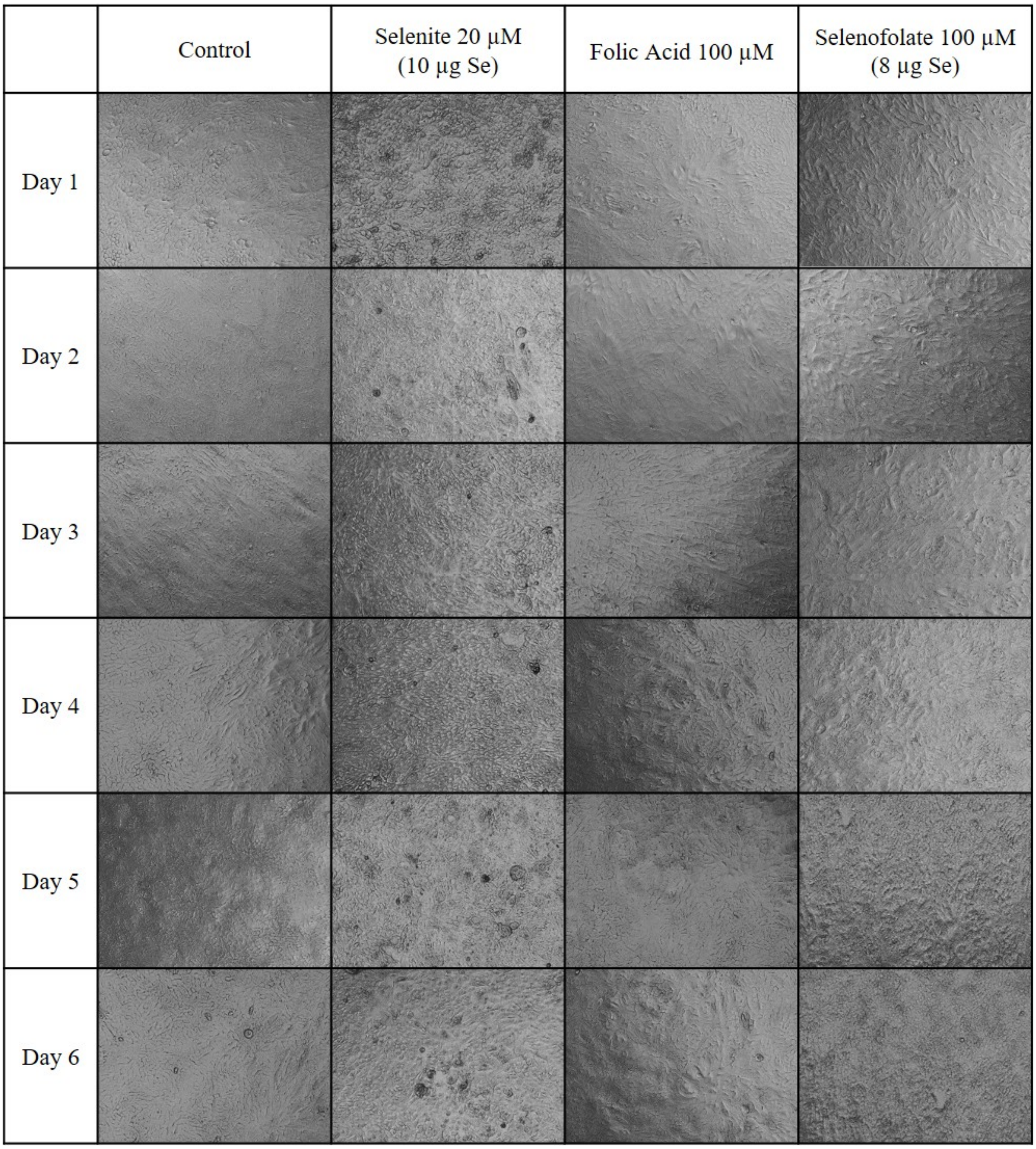

Figure S4. Photomicrographs of the morphological changes observed in HME50-5E cells. Cells were untreated (PBS Control) or treated with 20 µM Selenite (10 µg Se), 100 µM Folic Acid and 100 µM Selenofolate (8 µg Se) over 6 days. Treatment of HME50-5E cells with 20 µM Selenite and 100 µM Selenofolate did not induce severe morphological cell changes in comparison to those seen in the selenium-treated MDA-MB-468 cells. Representative fields of view of HME50-5E cells. Cells were photographed under phase contrast conditions at 20X magnification.

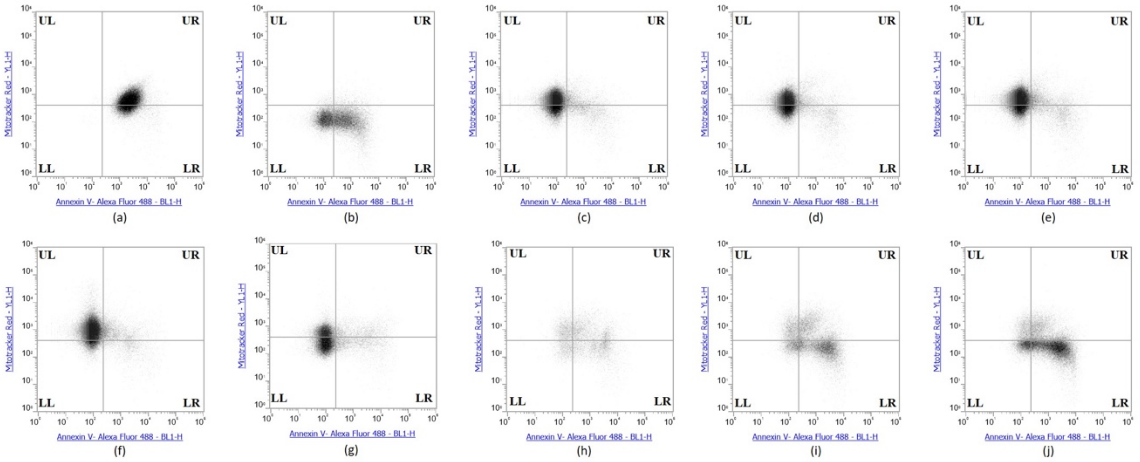

Figure S5. Folate, Selenofolate and Selenite as Se Treatments induced apoptosis in MDA-MB-468 Cells. (a) Sutent (apoptosis control), (b) H2O2 (necrosis control), (c) Folic Acid 50 µM, (d) Folic Acid 75 µM, (e) Folic Acid 100 µM, (f) Control untreated, (g) Selenite 4 µM (2 µg Se), (h) Selenofolate 50 µM (4 µg Se), (i) Selenofolate 75 µM (6 µg Se), (j) Selenofolate 100 µM (8 µg Se). MDA-MB-468 cells were stained with Annexin V/MitoTracker Red and subjected to flow cytometric analysis. The four quadrants represent—living cells (Upper Left; UL: Annexin V, MitoTracker Red), apoptotic (Upper Right; UR: Annexin V, MitoTracker Red), apoptotic (Lower Right; LR: Annexin V, MitoTracker Red), apoptotic (Lower Left; LL: Annexin V, MitoTracker Red).

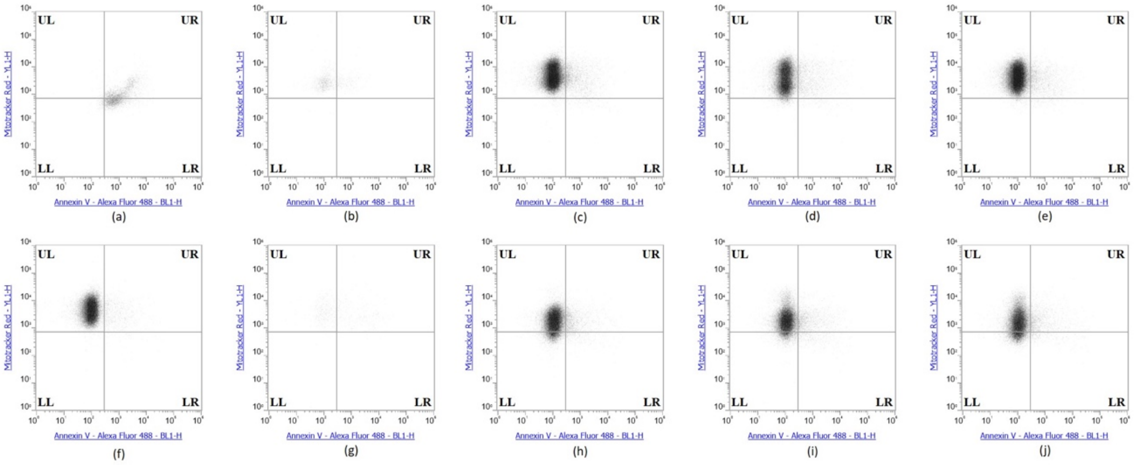

Figure S6. Selenofolate induced no apoptosis in HME50-5E Cells. (a) Sutent (apoptosis control), (b) H2O2 (necrosis control), (c) Folic Acid 50 µM, (d) Folic Acid 75 µM, (e) Folic Acid 100 µM, (f) Control untreated, (g) Selenite 4 µM (2 µg Se), (h) Selenofolate 50 µM (4 µg Se), (i) Selenofolate 75 µM (6 µg Se), (j) Selenofolate 100 µM (8 µg Se). HME50-5E cells were stained with Annexin V/MitoTracker Red and subjected to flow cytometric analysis. The four quadrants represent—living cells (Upper Left; UL: Annexin V, MitoTracker Red), (Upper Left; UL: Annexin V, MitoTracker Red), apoptotic (Upper Right; UR: Annexin V, MitoTracker Red), apoptotic (Lower Right; LR: Annexin V, MitoTracker Red), apoptotic (Lower Left; LL: Annexin V, MitoTracker Red).
